# Deep-Learning-Based Hepatic Ploidy Quantification Using H&E Histopathology Images

**DOI:** 10.3390/genes14040921

**Published:** 2023-04-16

**Authors:** Zhuoyu Wen, Yu-Hsuan Lin, Shidan Wang, Naoto Fujiwara, Ruichen Rong, Kevin W. Jin, Donghan M. Yang, Bo Yao, Shengjie Yang, Tao Wang, Yang Xie, Yujin Hoshida, Hao Zhu, Guanghua Xiao

**Affiliations:** 1 Quantitative Biomedical Research Center, Department of Population and Data Sciences, The University of Texas Southwestern Medical Center, Dallas, TX 75390, USA; 2 Children’s Research Institute, Departments of Pediatrics and Internal Medicine, Center for Regenerative Science and Medicine, The University of Texas Southwestern Medical Center, Dallas, TX 75390, USA; 3 Division of Digestive and Liver Diseases, Department of Internal Medicine, The University of Texas Southwestern Medical Center, Dallas, TX 75390, USA; 4 Center for the Genetics of Host Defense, The University of Texas Southwestern Medical Center, Dallas, TX 75390, USA; 5 Hamon Center for Regenerative Medicine, The University of Texas Southwestern Medical Center, Dallas, TX 75390, USA; 6 Department of Bioinformatics, The University of Texas Southwestern Medical Center, Dallas, TX 75390, USA; 7 Children’s Research Institute Mouse Genome Engineering Core, The University of Texas Southwestern Medical Center, Dallas, TX 75390, USA

**Keywords:** deep learning, hematoxylin-eosin (H&E) histopathology images, ploidy, liver

## Abstract

Polyploidy, the duplication of the entire genome within a single cell, is a significant characteristic of cells in many tissues, including the liver. The quantification of hepatic ploidy typically relies on flow cytometry and immunofluorescence (IF) imaging, which are not widely available in clinical settings due to high financial and time costs. To improve accessibility for clinical samples, we developed a computational algorithm to quantify hepatic ploidy using hematoxylin-eosin (H&E) histopathology images, which are commonly obtained during routine clinical practice. Our algorithm uses a deep learning model to first segment and classify different types of cell nuclei in H&E images. It then determines cellular ploidy based on the relative distance between identified hepatocyte nuclei and determines nuclear ploidy using a fitted Gaussian mixture model. The algorithm can establish the total number of hepatocytes and their detailed ploidy information in a region of interest (ROI) on H&E images. This is the first successful attempt to automate ploidy analysis on H&E images. Our algorithm is expected to serve as an important tool for studying the role of polyploidy in human liver disease.

## 1. Introduction

Polyploidy refers to the duplication of DNA content within a single cell. In general, polyploid cells contain more than two homologous sets of chromosomes, and this increase in genome copies can occur on the cellular level, nuclear level, or both. For example, an octoploid cell can contain either a single octoploid nucleus or two tetraploid nuclei. In this manuscript, we regard polyploidy as a combination of cellular ploidy (the number of nuclei per cell) and nuclear ploidy (the DNA content per nucleus).

Polyploidy appears in several tissues of vital functions, including the brain [1], heart [2], muscle [3], bone marrow [4,5], and liver [6,7,8]. Importantly, the liver contains a large number of polyploid hepatocytes. Up to 90% of rodent hepatocytes and 40% of human hepatocytes are polyploid [8,9,10], making this an extraordinary property of the liver. A major challenge in the field is to understand the biological impact of liver polyploidy. Dynamic changes in hepatic ploidy have been observed during liver disease progression [11,12,13,14]. For example, Toyoda et al. found that the fraction of polyploid hepatocytes positively correlates with severity of chronic hepatitis [11]. Gentric et al. discovered pathological polyploidization in nonalcoholic fatty liver disease (NAFLD), which is a pervasive hepatic metabolic disorder and has been identified as a risk factor for hepatocellular carcinoma (HCC) [12]. Liu et al. proposed to reverse the age-related function disorder of the liver by the conversion of hepatocyte ploidy [14]. However, the role of polyploid hepatocytes in liver development and disease is still unclear. Some studies suggest that polyploidization can prevent tumorigenesis associated with either acute mutagen exposure or chronic tissue damage [15,16,17,18]. Lin et al. found that polyploid hepatocytes readily contribute to tissue regeneration through proliferation, but are resistant to mitotic errors and loss of tumor suppressors [18]. The discovery of this physiologic adaptation suggests innovative ways to prevent liver cancer in high-risk cirrhotic patients. In contrast, other studies suggest that polyploidy can also promote the initiation of liver cancer [19,20,21]. Lin et al. found that the hyperpolyploidization of hepatocytes around the centrilobular (CL) region is closely linked with the development of HCC cells after diethylnitrosamine treatment [19]. Overall, the function of polyploid hepatocytes has not yet been fully understood and needs further exploration [22,23,24]. Therefore, the improved characterization of the frequency and number of polyploid cells in both normal and diseased human livers would be important.

Currently, the quantification of hepatic ploidy relies on two main technological approaches: flow cytometry and image-based analysis using immunofluorescence (IF) [12,15,16,17,18,19,25,26]. Flow cytometry [14,21,27,28,29] is the current gold standard for ploidy quantification. However, it requires fresh samples, which can be difficult to obtain in clinical settings. Flow cytometry also requires the enzymatic dissociation of those samples, which is very challenging for scarred fibrotic tissues. IF imaging [11,30] is often used to visualize hepatic ploidy. However, it can be difficult to obtain unstained tissue sections, and sampling errors may arise from manual counting in a few histological regions. In order to overcome the difficulties of flow cytometry and IF imaging, we proposed an automated system to quantify hepatic ploidy based on hematoxylin-eosin (H&E) histopathology images. H&E staining is the most widely used staining method in histological examination, and is routinely performed for the clinical evaluation of liver disorders [31,32]. The accurate assessment of hepatic ploidy using H&E slides would allow the analysis of existing clinical samples without further sectioning and immunostaining. Therefore, our proposed method will broaden the application of hepatic ploidy quantification to more patients, in addition to decreasing manual labor.

**Figure 1:**
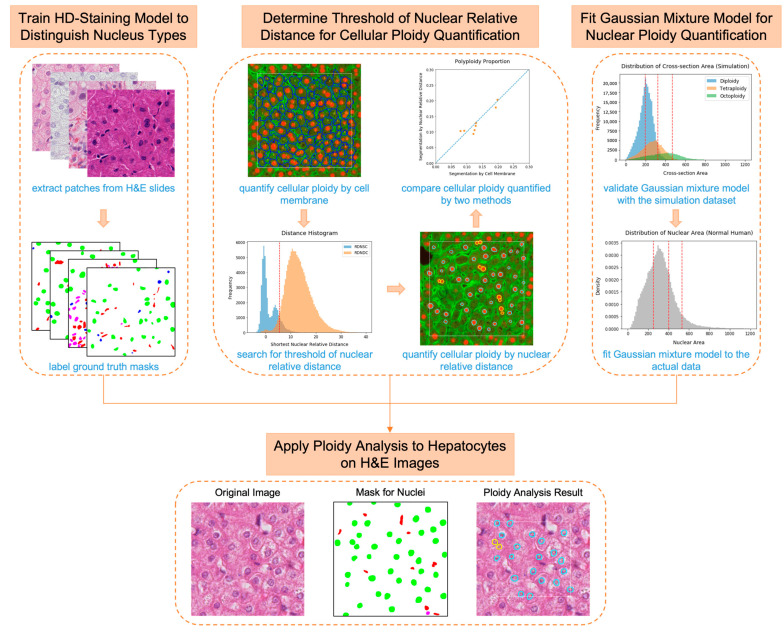
Development process of the automated hepatic ploidy quantification algorithm.

We developed an algorithm to measure hepatic ploidy on H&E images (Figure 1), which consists of three major steps: (1) distinguishing the nuclei of different cell types with the Histology-based Digital (HD)-Staining model [33]; (2) determining whether two nuclei are in the same cell or different cells by nuclear relative distance; (3) quantifying the DNA content within each single nucleus using the fitted Gaussian mixture model. In this manuscript, we will introduce each step of the algorithm in detail. For the first step, we will describe the development process of the HD-Staining model in Section 2.2 and demonstrate its performance in Section 3.1. For the second step, we will discuss selecting the threshold of nuclear relative distance in Section 2.3 and validating its reliability for cellular ploidy quantification in Section 2.4 and Section 3.2. For the third step, we will describe the fitting process of the Gaussian mixture model in Section 2.5 and validate its reliability for nuclear ploidy quantification in Section 3.3. In the end, we will show examples of the total ploidy analysis results of hepatocytes on H&E images in Section 3.4. Additionally, we will showcase our website, which carries out all of these functions, in Section 3.5.

The main contributions of this paper are:

- We developed the first automated algorithm to quantify hepatic ploidy based on H&E histopathology images;
- We trained a deep learning model to segment and classify cell nuclei in liver tissue;
- We overcame the difficulty of quantifying cellular ploidy in the absence of cell membrane information on H&E images. We proved the validity of using nuclear relative distance as a new standard to determine the relationship between neighboring hepatic nuclei;
- We built a Gaussian mixture model to quantify nuclear ploidy on H&E images and validated its reliability with a simulation dataset;
- We created a user-friendly website to facilitate the widespread use of this algorithm.

## 2. Materials and Methods

### 2.1. Data Collection

Eight 20× magnification hepatic IF slides (with 1 pixel equivalent to 0.65 μm) were used to calculate and validate the threshold of nuclear relative distance for cellular ploidy determination, including two normal mouse liver slides, four normal human liver slides, and two cirrhotic human liver slides. All slides were stained with HNF4A to highlight hepatocyte nuclei and CTNNB1 to highlight the cell membrane. Tissue samples were fixed overnight in 4% paraformaldehyde (PFA; Alfa Aesar, Tewksbury, MA, USA, #J19943K2) at 4 °C. Fixed tissues were embedded in paraffin and sectioned by the UTSW Histopathology Core. The paraffin-sectioned slides were deparaffinized in xylene and rehydrated in 100%, 90%, 80%, 70%, 50%, and 30% ethanol and deionized water. Citra Plus Antigen Retrieval (Fisher Scientific, Waltham, MA, USA, #NC9755543) with 0.05% Tween 20 was used for heat-induced antigen retrieval by microwaving. After a cool down, the slides were blocked in 5% BSA with 0.25% Triton X-100 at room temperature for 1 h. A Mouse-On-Mouse Immunodetection Kit (M.O.M Kit; Fisher Scientific, Waltham, MA, USA, #NC9764536) was further used for staining according to the manufacturer’s protocols. The slides were incubated with HNF4A (Santa Cruz, Dallas, TX, USA, #sc-8987) and CTNNB1 (BD Biosciences, Franklin Lakes, NJ, USA, #610154) at a 1:200 dilution in the blocking solution from M.O.M Kit at 4 °C overnight. After washing three times with PBST, slides were incubated with Alexa Fluor 594 donkey anti-rabbit IgG antibody (Life technologies, Carlsbad, CA, USA, #A-21207) and Alexa Fluor 488 goat anti-mouse IgG1 antibody (Life technologies, Carlsbad, CA, USA, #A-21121) at a 1:200 dilution and Hoechst 33,342 (Life technologies, Carlsbad, CA, USA, #H3570) at a 1:1000 dilution in the blocking solution from M.O.M Kit at room temperature for 1 h. Whole slides were imaged by an Axioscan slide scanner (Zeiss, Jena, Germany) in the UTSW Whole Brain Microscopy Facility and processed using Zen 2.6 software from Zeiss (Jena, Germany).

In addition to IF slides, a total of seventy-six 40× magnification hepatic H&E slides (with 1 pixel equivalent to 0.23 μm) were collected from two different sources. The first set of slides, containing two normal mouse liver slides, nine normal human liver slides, and two cirrhotic human liver slides without HCC, were subjected to standard H&E staining by the UTSW Histopathology Core and imaged by a Hamamatsu Nanozoomer 2.0HT (Hamamatsu Photonics, Hamamatsu, Japan) in the UTSW Whole Brain Microscopy Facility. The second set of slides, containing sixty-three cirrhotic human liver slides with HCC, was obtained from our previous study (PMID: 18923165). Under the supervision of a board-certified gastrointestinal (GI) pathologist, HCC patient slides were annotated for the non-malignant regions, defined as regions of interest (ROIs) in this study.

For assessing the hepatic ploidy of normal humans by flow cytometry, the Human Suspension Hepatocytes (male, single donor and metabolism qualified, Fisher Scientific, Waltham, MA, USA, #HMCS1S) were thawed according to the manufacturer’s protocols. After thawing, the cells were fixed in 75% ethanol at −20 °C. For the detection of ploidy populations, the cells were washed with 1% BSA in PBS for three times, then incubated with PI/RNase Staining Buffer (BD Biosciences, Franklin Lakes, NJ, USA, #550825) at 25 °C for 15 min. Cells were analyzed with a BD FACS Aria Fusion machine (BD Biosciences, Franklin Lakes, NJ, USA).

### 2.2. Nuclei Segmentation and Classification on H&E Images Using the HD-Staining Model

Since hematoxylin stains all cell nuclei non-selectively, we needed a way to exclusively focus on hepatocytes while excluding other cells. The Mask Regional Convolutional Neural Network (Mask-RCNN) is the state-of-the-art architecture for instance segmentation tasks [34,35]. The HD-Staining model [33] is an implementation of Mask-RCNN architecture in the analysis of H&E images. It has demonstrated high accuracy in the segmentation and classification of cell nuclei in lung tissue. Therefore, we decided to adapt the HD-Staining algorithm for nuclei detection in liver tissue by transfer learning [36,37] using a newly prepared H&E stained liver tissue imaging dataset. This dataset consisted of fifty image patches (500 × 500 pixels) randomly extracted from the seventy-six hepatic H&E slides. On these image patches, nuclei were manually labelled under the supervision of the board-certified GI pathologist. These manually annotated images were used as the ground truth masks, where each pixel was specified to one of seven categories: hepatocyte nuclei, stroma nuclei, lymphocyte nuclei, macrophage nuclei, red blood cells, karyorrhexis, and background. These fifty image patches and their corresponding ground truth masks were then randomly assigned to the training set, validation set, or testing set at a ratio of 8:1:1. Over two thousand nuclei of different cell types were involved in the training process. To further increase model generalizability and accuracy, several manipulations were performed on the training set at the pre-processing stage. Firstly, the signal in each RGB channel was transformed into a variable on a standard normal distribution and then randomly shifted by linear transformation [38]. Secondly, random image augmentations, such as flip and projective transformations, were applied to all image patches in the training set and their masks in step. Processed images were fed into a pretrained HD-Staining neural network, and trained with a learning rate of 0.01 and a momentum of 0.9. The learning rate was set to 0.01 because it is a relatively small value and is commonly used as a default or starting point for many deep learning models [39]. The momentum of 0.9 was used to accelerate the gradient-based optimization process [40]. The model with the best performance in the validation set was selected. In order to match the nuclei detected by the selected model to the nuclei on the ground truth masks, the Intersection over Union (IoU) [41] between each predicted nucleus and each ground truth nucleus was calculated individually. The matched ground truth nucleus was defined as the one with maximum IoU for a specific predicted nucleus. Since the HD-Staining model can generate bounding box, mask, and class simultaneously, accuracies of segmentation and classification were both evaluated. For all nuclei on the ground truth masks, the percentage of coverage was used to measure the model sensitivity for nuclei detection. For the matched nuclei, the IoU was calculated to show segmentation performance and a paired comparison of classification accuracy was depicted by the confusion matrix.

### 2.3. Thresholding for Nuclear Relative Distance

**Figure 2:**
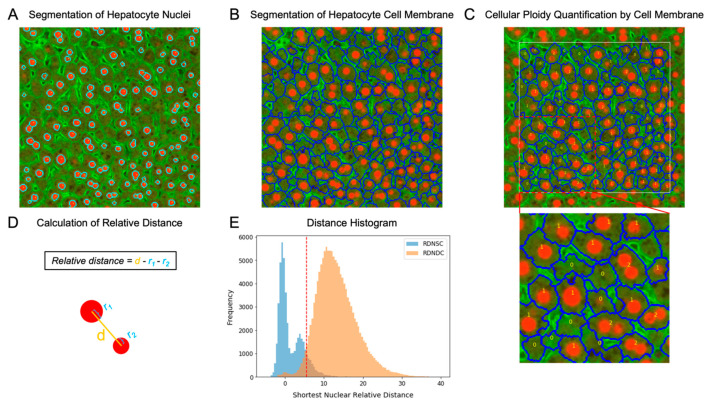
Calculation of nuclear relative distance threshold. (**A**) Nuclei segmentation by watershed. The nuclear boundary is labelled with the cyan line, and the centroid of each nucleus is marked in yellow. (**B**) Cell membrane segmentation by watershed. The cellular boundary is labelled with the blue line. (**C**) Cellular ploidy quantification using cell membrane information. The number indicates the cellular ploidy of the target cell. The white square demonstrates the central region for analysis. The detailed cellular ploidy quantification result of a small region (150 × 150 pixels) is also shown below. (**D**) The diagram illustrates the calculation of relative distance between nuclei. (**E**) The histogram shows the distribution of RDNSC (relative distance between nuclei within the same cell) and RDNDC (relative distance between nuclei within different cells). The dotted red line indicates the selected ${threshold}_{IF}$ (5.5 pixels).

Since H&E images cannot provide valid cell membrane information, IF images were utilized to seek an appropriate threshold for distinguishing relative distance between nuclei within the same cell (RDNSC) and relative distance between nuclei within different cells (RDNDC), which would then be transformed to the threshold of H&E images in proportion to the spatial resolution. To speed up analysis, the tissue region of each IF slide was cropped into small image patches (360 × 360 pixels). For individual image patches, the nuclei and cell membrane of hepatocytes were segmented by watershed [42], an effective image processing method, based on the signal intensities of HNF4A and CTNNB1, respectively (Figure 2A,B). By comparing the location of the nuclear centroid and cellular boundary, each nucleus was assigned to a specific cell. To avoid incomplete information of cells at the edge of small image patches, only the cells whose centroid was in the central region (300 × 300 pixels) and their nuclei were considered in the following steps (Figure 2C).

The relative distance between any pair of hepatocyte nuclei appearing on the same IF patch was automatically calculated according to the formula:

(1) $$relative\;distance=d-r_{1}-r_{2}$$

where d indicates the absolute distance between nuclear centroids, and $r_{1}$ and $r_{2}$ indicate the radius of each nucleus (Figure 2D). The absolute distance (d) was measured by the “kneighbors_graph” function of the scikit-learn package (version 0.19.1) [43], and the nuclear radius (r) was measured by the “regionprops” function of the scikit-image package (version 0.15.0) [44]. For all nuclei, the relative distance between the target nucleus and its closest nucleus in a different cell was collected (RDNDC). In addition, for nuclei in polynuclear cells, the relative distance between the target nucleus and its closest nucleus within the same cell was collected (RDNSC). Distribution histograms of RDNDC and RDNSC were plotted, and a ${threshold}_{IF}$ (5.5 pixels) of nuclear relative distance was obtained to maximize the F1 score (Figure 2E). The cutoff value of H&E images, ${threshold}_{HE}$ (15.54 pixels), was transformed from ${threshold}_{IF}$ according to their spatial resolution difference (0.65 μm/pixel for IF images and 0.23 μm/pixel for H&E images):

(2) $${threshold}_{HE}=\frac{{threshold}_{IF}\times {resolution}_{IF}}{{resolution}_{HE}}$$

### 2.4. Hepatocyte Cellular Ploidy Determination by Nuclear Relative Distance

The IF and H&E slides were cropped into small image patches for ploidy quantification (360 × 360 pixels for IF patches and 500 × 500 pixels for H&E patches). By applying the watershed to IF patches or the HD-Staining model to H&E patches, masks specific for hepatocyte nuclei were extracted. The relative distance between any pair of hepatocyte nuclei on the same image patch was calculated based on their centroid locations and nuclear radii (Figure 2D). By comparing their relative distance with the predetermined threshold value, we could determine whether two nuclei were more likely to be in the same hepatocyte. The cell location was defined as the arithmetic average of centroid coordinates of all nuclei within it. Only the cells whose centroid was in the central region (300 × 300 pixels for IF patches and 330 × 330 pixels for H&E patches) were preserved, in case of incomplete information at the image edge. As a result, the total hepatocyte number as well as the nucleus number in each hepatocyte could be summarized for each ROI.

### 2.5. Hepatocyte Nuclear Ploidy Quantification by Nuclear Area

#### 2.5.1. Establishment of the Simulation Dataset

Based on the ploidy information of normal human hepatocytes from previous studies [9,30], all parameters of the simulated model were selected to mimic the real case. The numbers of diploid, tetraploid, and octoploid nuclei were set to be 360,000, 122,500, and 62,500, which, respectively, contributed 66.05%, 22.48%, and 11.47% of the total. For a certain type of nuclei, we assumed that the spherical radii followed a normal distribution, and that the distances from the spherical center to the cross-section followed a uniform distribution. Taking diploid nuclei as the example, the mean ($\mu_{di}$) was set as 9, and standard deviation ($\sigma_{di}$) was calculated by:

(3) 3×σ=a×μ

where a was determined to be 0.3 by fitting the distribution of the observed data. Therefore, 600 spherical radius values were randomly simulated from a normal distribution with a mean ($\mu_{di}$) of 9 and a corresponding standard deviation ($\sigma_{di}$) of 0.9 (Figure S1B). Then, for a diploid sphere of a fixed radius, 600 cross-sections were created by cutting along the spherical central axis randomly with equal probability. In total, 360,000 cross-section areas of diploid nuclei were obtained. Since it is difficult to detect small nuclei in practice, cross-sections with areas smaller than 200 were dropped proportionally. The same process was applied to tetraploid nuclei and octoploid nuclei. In theory, the volume of diploid nuclei, tetraploid nuclei, and octoploid nuclei should increase by two multipliers, which means:

(4) $$\mu_{tetra}=\sqrt[{3}]{2}\times \mu_{di},\;\mu_{octo}=\sqrt[{3}]{2}\times \mu_{tetra}$$

However, considering that there is only one obvious peak in the nuclear area distribution of normal human hepatocytes (Figure S1H), we gradually decreased the value of the multiplier from $\sqrt[{3}]{2}$ (1.26) to search for the value when three histograms merge together, which was 1.18. Therefore, in our simulation data (Figure S1C,D):

(5) $$\mu_{tetra}=1.18\times \mu_{di},\;\mu_{octo}=1.18\times \mu_{tetra}$$

The simulation data for all categories were plotted (Figure S1E) and gathered for model fitting (Figure S1F).

#### 2.5.2. Gaussian Mixture Model Fitting and Predicting Process

The Gaussian mixture model was fitted to the simulation data or real data using the built-in functions of the scikit-learn package (version 0.19.1) [43] in Python (version 3.6.6). The fitting process started from a Gaussian mixture model of only one component to locate the position of the most obvious peak (k). After obtaining the value of k, another Gaussian mixture model of three components provided with the initial means (k, k×1.4, $k\times 1.4^{2}$) was fitted to the data. Using the model built based on hepatocyte nuclear areas detected on eight normal human liver H&E slides, the ploidy of hepatocyte nuclei on other human H&E slides would be automatically classified. The nuclei predicted as belonging to the first mixture component of the fitted Gaussian mixture model would be regarded as diploid nuclei, the nuclei of the second mixture component would be regarded as tetraploid nuclei, and the nuclei of the third mixture component would be regarded as octoploid nuclei.

## 3. Results

### 3.1. The HD-Staining Model Recognized Hepatocyte Nuclei on H&E Images

**Figure 3:**
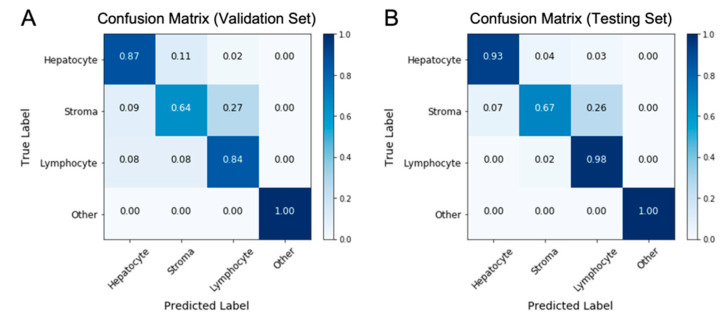
The HD-Staining model for nuclei segmentation and classification on hepatic H&E images. The performance of the HD-Staining model is shown by the confusion matrices between the predicted label and true label in the validation set (**A**) and testing set (**B**). The classification accuracies of the macrophage nuclei, red blood cells, and karyorrhexis are included in the “other” category of the confusion matrices.

Using the HD-Staining algorithm for liver tissue trained via transfer learning, nuclei on hepatic H&E images could be automatically segmented and classified into six categories except for background: hepatocyte, stroma cell, lymphocyte, macrophage, red blood cell, and karyorrhexis. Three criteria were used to evaluate the performance of the model: coverage, IoU, and confusion matrix. The coverage percentage of all nuclei was 93.48% in the validation set and 88.22% in the testing set. For hepatocytes, the IoU of the detected hepatocyte nuclei appeared to be 82.10% in the validation set and 82.17% in the testing set. According to the confusion matrix (Figure 3), the classification accuracy of hepatocyte nuclei was 86.67% in the validation set and 93.16% in the testing set. In summary, the HD-Staining algorithm for liver tissue performed well in distinguishing hepatocyte nuclei from other nuclei on H&E images.

### 3.2. Nuclear Relative Distance Determined Hepatic Cellular Ploidy

**Figure 4:**
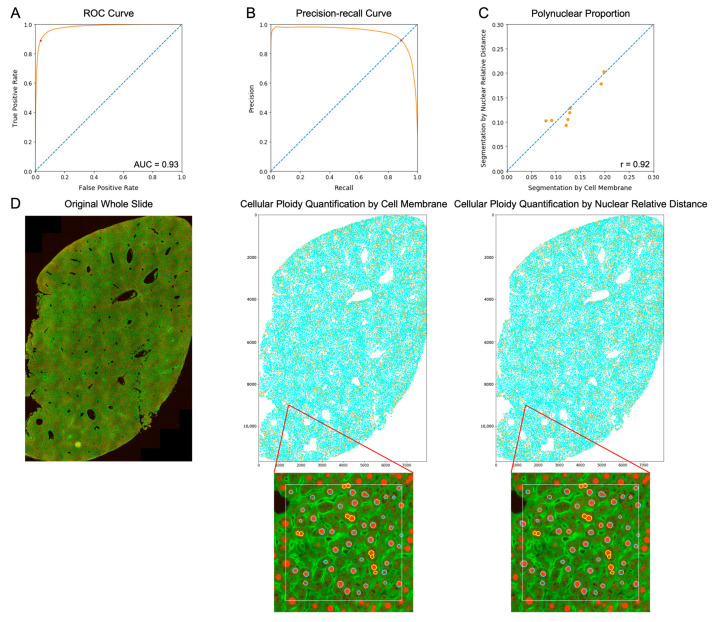
Evaluation of the accuracy of cellular ploidy determined by nuclear relative distance. (**A**) The ROC curve of cellular ploidy determined by nuclear relative distance with an AUC of 0.93. The ground truth is the cellular ploidy quantification using cell membrane information. The selected ${threshold}_{IF}$ (5.5 pixels) is denoted by the red dot. (**B**) The precision-recall curve of cellular ploidy determined by nuclear relative distance. The selected ${threshold}_{IF}$ (5.5 pixels) is denoted by the red dot. (**C**) The scatter plot demonstrates the high correlation of polynuclear proportion between cell membrane segmentation and nuclear relative distance segmentation. (**D**) The distribution maps of mononuclear cells and polynuclear cells determined by cell membrane or nuclear relative distance. Cyan dots correspond to mononuclear cells, and yellow dots correspond to polynuclear cells. The detailed cellular ploidy quantification result of a small region (300 × 300 pixels) is shown below the distribution map, where nuclei within mononuclear cells are marked in cyan, and nuclei within polynuclear cells are marked in yellow.

Since the cell membrane is not clearly visualized in the H&E images, another method to determine whether two nuclei are in the same or different cells was needed. For this purpose, the relative distance between nuclei was used as a new indicator to identify nuclear relationship. To find the threshold of nuclear relative distance from IF images, the true positive, false positive, true negative, and false negative at each threshold point were calculated using the cellular ploidy quantification result by cell membrane as the ground truth, and the receiver operating characteristic (ROC) curve was drawn (Figure 4A). The area under the ROC curve (AUC) was 0.93. Moreover, the precision-recall curve, labelled by a red dot corresponding to the selected cutoff value, showed that the F1 score was maximized at the ${threshold}_{IF}$ of 5.5 pixels (Figure 4B).

In order to evaluate the performance of our new cellular ploidy determination method, the cell membrane or ${threshold}_{IF}$ was used to determine the relationship between hepatocyte nuclei on eight IF slides. Consequently, the number of nuclei in each hepatocyte was clear. The polynuclear proportions identified by these two methods agreed with a correlation coefficient of 0.92 (Figure 4C). Furthermore, to visualize the segmentation result for a whole slide, the distribution maps of mononuclear cells and polynuclear cells were drawn, which showed similar patterns between these two approaches (Figure 4D).

### 3.3. Nuclear Area Determined Hepatic Nuclear Ploidy

Based on the image information of individual nuclei, area is the most direct and reliable parameter for nuclear ploidy quantification. However, because most hepatocyte nuclei in the normal human liver are diploid, the histogram of nuclear area distribution only has one obvious peak (Figure S1H), making it difficult to distinguish the three most common types of nuclear ploidy (diploidy, tetraploidy, and octoploidy) directly from H&E images. A dataset was built to simulate the area distribution of diploid, tetraploid, and octoploid hepatocyte nuclei according to their size and proportion in the normal human liver (Figure S1A). In the simulation dataset, the histogram of estimated areas showed a distribution similar to that in the observed dataset (Figure S1F). A Gaussian mixture model of three components was fitted to the simulation data. The histogram shown in Figure S1G demonstrated that the fitted model estimated the mean of each simulated mixture component reasonably well. The Gaussian mixture model of similar structure was then fitted to the observed data from eight normal human liver H&E slides (Figure S1H) and used for the following nuclear ploidy determination.

### 3.4. Total Ploidy Analysis of Hepatocytes on Human H&E Images

**Figure 5:**
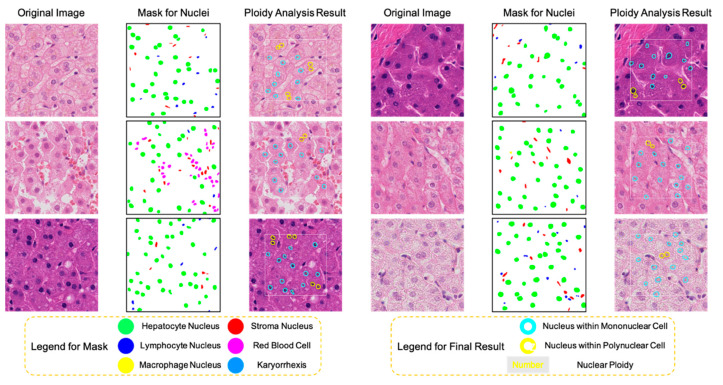
Deep-learning-based hepatic ploidy analysis of human H&E images.

**Figure 6:**
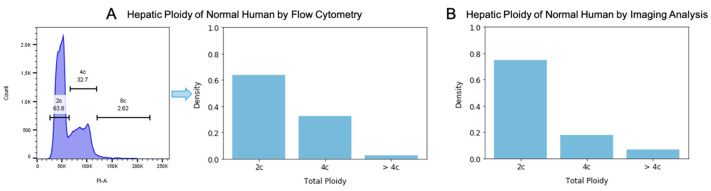
Comparison of the hepatic ploidy quantification result by flow cytometry and imaging analysis. (**A**) The hepatic ploidy profile of a normal human by flow cytometry. (**B**) The hepatic ploidy profile of a normal human by imaging analysis based on the H&E slide.

For the hepatic ploidy analysis of H&E images, the hepatocyte nuclei were recognized and localized by the HD-Staining model. Next, hepatic cellular ploidy was determined by calculating the relative distance between each pair of hepatocyte nuclei on the same image and comparing its value to ${threshold}_{HE}$ (15.54 pixels). Then, for each hepatocyte nucleus, nuclear ploidy was assessed by the Gaussian mixture model fitted on the nuclear area distribution of normal human hepatocytes. Taking cellular ploidy and nuclear ploidy together, the total ploidy for individual hepatocytes was measured. Figure 5 showed examples of hepatic ploidy analysis results on different H&E images. Figure 6B showed the total ploidy distribution of hepatocytes on an independent normal human liver H&E slide, which was not involved in the training set. The ratio of each ploidy category acquired from imaging analysis was consistent with the result from flow cytometry, demonstrating the reliability of our method (Figure 6A,B).

### 3.5. Online Implementation of Hepatic Ploidy Quantification on Human H&E Images

A website, https://lce.biohpc.swmed.edu/icpq/ (accessed on 11 April 2023), was developed to allow public usage of the hepatic ploidy analysis algorithm (Figure S2). This online tool takes a 40× H&E patch as the input. Users can adjust two parameters to make the analysis flexible: (1) ${threshold}_{HE}$, the threshold of nuclear relative distance (with a default value of 15.54), and (2) ${padding}_{HE}$, the margin around the central region of the target image patch (with a default value of 85). The output consists of two resulting images displayed on the web page and a downloadable comma-separated values (CSV) file. One image shows the segmentation and classification result by the HD-Staining model, and the other shows the ploidy quantification result specific for hepatocytes (Figure S3A). The output CSV file contains detailed information of each hepatocyte, including cell location, cellular ploidy, nuclear ploidy, total ploidy, individual nuclear area, and nuclear ploidy probability (Figure S3B). The nuclear ploidy probability is calculated with our Gaussian mixture model.

## 4. Discussion

In this study, we developed a deep-learning-based quantification algorithm to measure hepatic ploidy on standard H&E images. To the best of our knowledge, this is the first algorithm that can quantify ploidy on H&E images. As a routine histological examination, H&E can reveal a considerable amount of information, but it is generally considered challenging to collect ploidy information directly from H&E images for the following reasons. Firstly, since hematoxylin stains the nuclei of all cells, the nuclei of other cells may interfere with ploidy counting in hepatocytes. In this case, nucleus types can only be differentiated by morphological features, which was successfully achieved by the HD-Staining model adapted to liver tissue. Our developed HD-Staining model was capable of efficiently and accurately identifying hepatocyte nuclei, stroma nuclei, lymphocyte nuclei, macrophage nuclei, red blood cells, karyorrhexis, and background. It allowed ploidy analysis to focus on the hepatocyte nuclei. Secondly, cell borders are too obscure to be delineated on H&E images, making it difficult to identify cell region and cell content. Therefore, a benchmark is required to determine the relationship of neighboring nuclei in the absence of cell membrane information. We proved that the nuclear relative distance was reliable for this task. Thirdly, there is uncertainty when quantifying nuclear ploidy based only on nuclear area, which was resolved by validating the model on a simulation dataset. We showed that our developed Gaussian mixture model competently identified hepatic nuclear ploidy. After overcoming these three challenges, we could obtain the hepatic ploidy profile from H&E images. Moreover, the classification of nuclei from the five other cell types could be useful for future research involving liver biology.

Recently, Bou-Nader et al. found that polyploidy is a new marker in liver disease and liver cancer classification [30], providing evidence for a functional relationship between hepatic ploidy and liver disease status. Nevertheless, their ploidy profile was obtained by manual counting in several liver sections stained by IF, which is time-consuming and subjective. In contrast, our trained algorithm is an entirely automated, objective pipeline for the high-throughput processing of images. In addition to IF imaging, flow cytometry [14,21,27,28,29] and image cytometry [45,46,47] are frequently utilized in experimental research to accurately quantify ploidy. However, these methods have not seen widespread use in clinic because it is difficult to perfuse and obtain viable hepatocytes from biopsies. Different from IF imaging or cytometry, H&E staining on standard histology slides is low-cost and stable, making it the most popular method for research and clinical use [32]. There is obvious value in the ploidy analysis of H&E images. Saini et al. tried to train a deep learning model to automatically detect polyploid giant cancer cells (PGCCs) on breast cancer H&E slides [48]. Their algorithm treated ploidy measurement as a classification task. On the other hand, our developed algorithm can calculate detailed ploidy for each single cell and therefore provide users with more information. To make our newly developed technique highly accessible in clinical practice, we also created a user-friendly website to effortlessly execute hepatic ploidy analysis on human H&E images. The ploidy profile would be summarized in a downloadable CSV file and visualized with labelled images. With this technology, one can test more hypotheses about the relationship between hepatic ploidy and liver disease.

However, there are limitations to this ploidy quantification method. Firstly, our HD-Staining model was trained on normal and cirrhotic liver tissue. When hepatocytes progress to tumor cells, the morphology of their nuclei will go through tremendous changes, making it difficult for our model to identify nuclei in liver tumor cells. In order to perform ploidy measurements in liver tumor regions rather than chronic disease regions, another category of liver tumor nuclei will need to be added to the current model. Secondly, in this study, we mainly focused on developing an algorithm for hepatic ploidy quantification. In the future, we wish to apply this tool to datasets of patients at different stages of liver disease to study changes in hepatic ploidy during disease progression. We can even try to adapt this algorithm to analyze ploidy of other types of tissue.

## 5. Conclusions

To summarize, the algorithm developed in this study can be used to quantify hepatic ploidy from H&E images. To make this algorithm more accessible, we have developed a website that implements hepatic ploidy analysis on human H&E images. This publicly accessible tool is anticipated to benefit future studies on hepatic ploidy.

## Data Availability

Data are available upon request.

## Supplementary Materials

The following supporting information can be downloaded at: https://www.mdpi.com/article/10.3390/genes14040921/s1. Figure S1: Illustration of the Gaussian mixture model fitting process for nuclear ploidy assessment; Figure S2: Website for hepatic ploidy quantification on human H&E images; Figure S3: Example results from our online hepatic ploidy quantification tool.
