# Supplementary material for: Deep-Learning-Based Hepatic Ploidy Quantification Using H&E Histopathology Images
Source: Genes (Basel). 2023 Apr 16;14(4):921. doi: 10.3390/genes14040921 (PMC10137944; doi:10.3390/genes14040921)
Supplement: Supplementary file 1 [file genes-14-00921-s001.zip › genes-2286889-supplementary.pdf]

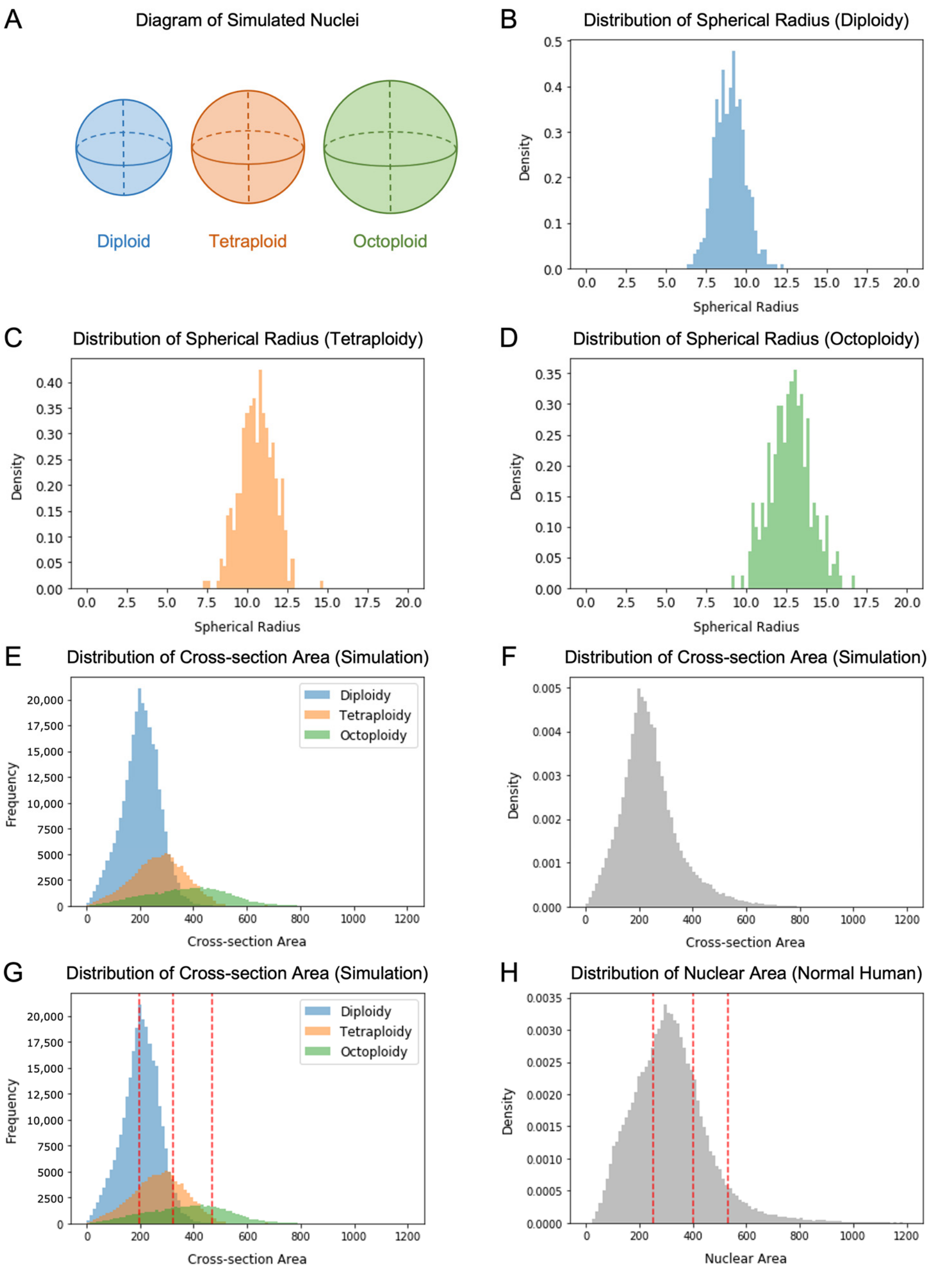

Figure S1. Illustration of the Gaussian mixture model fitting process for nuclear ploidy assessment. (**A**) A diagram of the scale of simulated nuclei across three categories. (**B-D**) The distributions of spherical radius of simulated diploid, tetraploid, or octoploid nuclei. (**E**) A histogram of the distribution of cross-section area of simulated diploid, tetraploid, and octoploid nuclei. (**F**) The distribution of all cross-section areas by mixing the data from three categories. (**G**) The resulting means after fitting a Gaussian mixture model to the mixed simulated cross-section areas, labelled with dotted red lines. (**H**) The resulting means after fitting a Gaussian mixture model to hepatocyte nuclear areas detected on eight normal human liver H&E slides, labelled with dotted red lines.

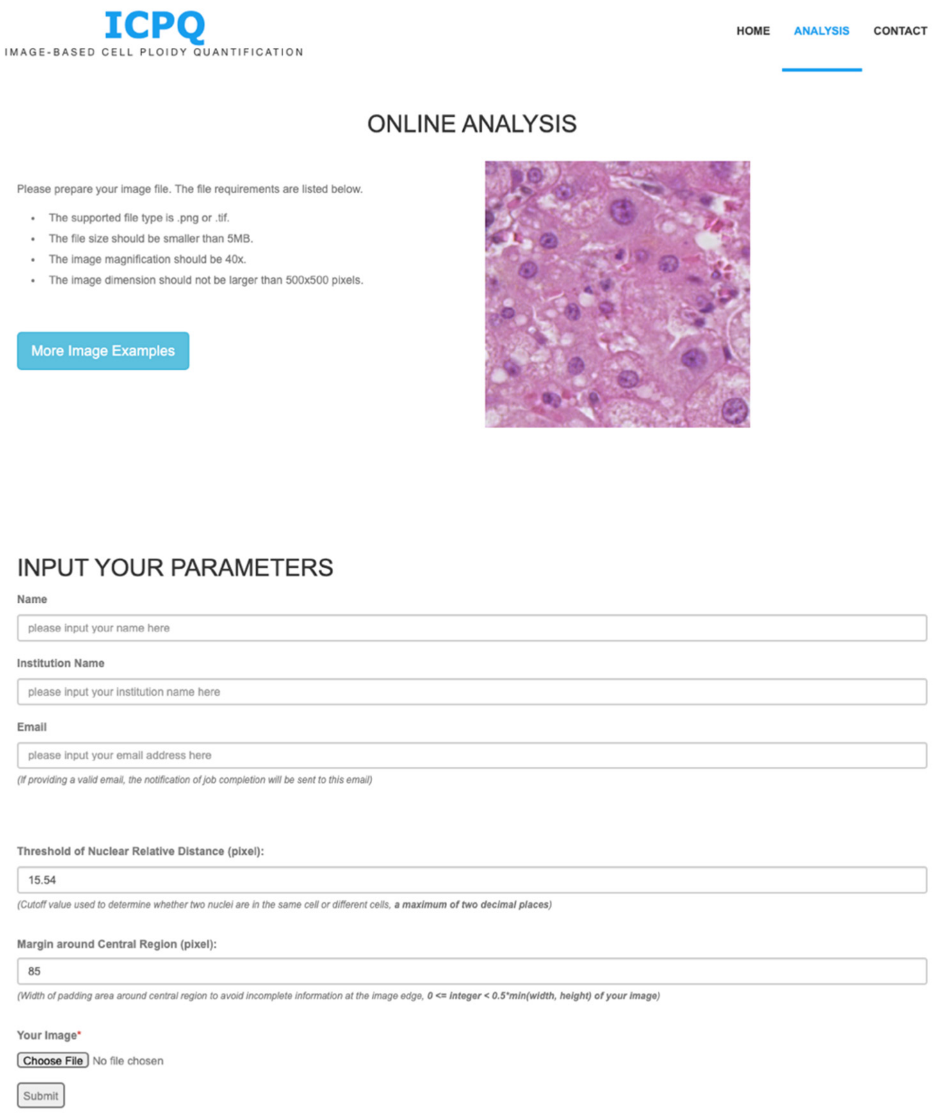

Figure S2. Website for hepatic ploidy quantification on human H&E images.

A

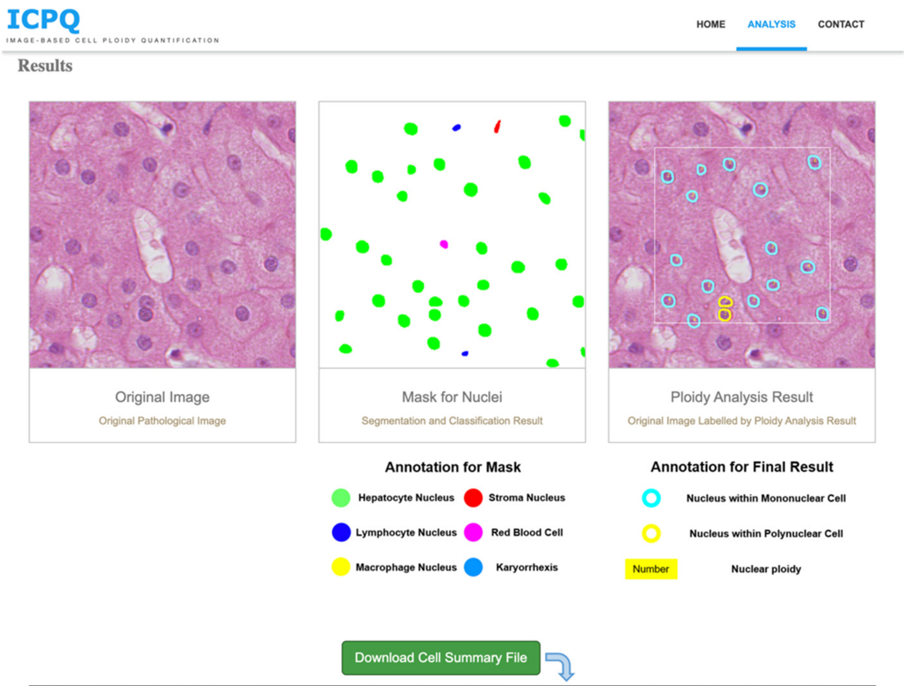

B

Cell id	Cell location	Cellular ploidy	Nuclear ploidy	Total ploidy	Nuclear area	Nuclear ploidy probability (2, 4, 8)
1	[113, 385]	1	4	4	425	(0.26, 0.64, 0.1)
2	[116, 225]	1	2	2	367	(0.5, 0.44, 0.06)
3	[126, 172]	1	2	2	203	(0.97, 0.01, 0.03)
4	[140, 109]	1	2	2	365	(0.51, 0.43, 0.06)
5	[164, 284]	1	4	4	489	(0.12, 0.65, 0.23)
6	[176, 155]	1	2	2	280	(0.88, 0.08, 0.03)
7	[274, 305]	1	2	2	348	(0.6, 0.34, 0.05)
8	[297, 125]	1	2	2	309	(0.79, 0.17, 0.04)
9	[310, 373]	1	4	4	420	(0.27, 0.63, 0.09)
10	[343, 307]	1	2	2	320	(0.74, 0.21, 0.04)
11	[346, 185]	1	2	2	371	(0.48, 0.46, 0.06)
12	[373, 111]	1	4	4	430	(0.24, 0.65, 0.1)
13	[373, 270]	1	2	2	335	(0.67, 0.28, 0.05)
14	[387, 217]	2	2, 2	4	336, 364	(0.67, 0.28, 0.05), (0.52, 0.42, 0.06)
15	[398, 400]	1	4	4	387	(0.4, 0.53, 0.07)
16	[411, 158]	1	4	4	409	(0.31, 0.6, 0.08)

Figure S3. Example results from our online hepatic ploidy quantification tool. (**A**) The result page displays the mask for nuclei output from the HD-Staining model and the ploidy analysis result for hepatocytes. (**B**) A downloadable CSV file provides detailed information for each hepatocyte, including cell location, cellular ploidy, nuclear ploidy, total ploidy, individual nuclear area, and estimated nuclear ploidy probability.
